# Identification of circulating microRNA signatures as potential noninvasive biomarkers for prediction and prognosis of lymph node metastasis in gastric cancer

**DOI:** 10.18632/oncotarget.17789

**Published:** 2017-05-10

**Authors:** Xiumei Jiang, Wenfei Wang, Yongmei Yang, Lutao Du, Xiaoyun Yang, Lili Wang, Guixi Zheng, Weili Duan, Rui Wang, Xin Zhang, Lishui Wang, Xiaoyang Chen, Chuanxin Wang

**Affiliations:** ^1^ Department of Clinical Laboratory, The Second Hospital of Shandong University, Jinan, 250033, Shandong Province, China; ^2^ Humanistic Medicine Research Center, Shandong University, Jinan, 250012, Shandong Province, China; ^3^ Department of Clinical Laboratory, Qilu Hospital, Shandong University, Jinan, 250012, Shandong Province, China

**Keywords:** microRNA, lymph node metastasis, gastric cancer, prediction, prognosis

## Abstract

Circulating microRNAs (miRNAs) are emerging as novel noninvasive biomarkers for prediction of lymph node metastasis (LNM) in cancer. The aim of this study was to identify serum miRNA signatures for prediction and prognosis of LNM in gastric cancer (GC). MiSeq sequencing was performed for an initial screening of serum miRNAs in 10 GC patients with LNM, 10 patients without LNM and 10 healthy controls. Reverse transcription quantitative real-time PCR was applied to confirm concentration of candidate miRNAs using a training cohort (*n =* 279) and a validation cohort (*n =* 180). We identified a four-miRNA panel (miR-501-3p, miR-143-3p, miR-451a, miR-146a) by multivariate logistic regression model that provided high predictive accuracy for LNM with an area under the receiver operating characteristic curve (AUC) of 0.891 (95% CI, 0.840 to 0.930) in training set. Prospective evaluation of this panel revealed an AUC of 0.822 (95% CI, 0.758 to 0.875, specificity = 87.78%, sensitivity = 63.33%) in validation set. Moreover, Kaplan–Meier analysis showed that LNM patients with low miR-451a and miR-146a levels had worse overall survival (OS) (*p* < 0.05). In Cox regression analysis, miR-451a was independently associated with OS of LNM (*p* = 0.028). Our results suggested that use of serum miRNAs seems promising in estimating the probability GC patients harbor LNM and providing prognostic information for LNM.

## INTRODUCTION

Gastric cancer (GC) is the fourth most common malignancy and the second most frequent cause of cancer-related death worldwide, with an estimated 951,600 new cases and 723,100 deaths in 2012 [1]. About 50–75% of GC patients at stage III or IV display lymph node metastasis (LNM) and the overall incidence of LNM in early stage patients ranges from 5% to 20% [2–4]. Presence of LNM is one of the most important prognostic factors and lymph node dissection has been proved to improve GC survival [5]. Preoperative determination of lymph node status is critical in providing guide on tumor staging and planning optimal treatment for GC. To date, imaging techniques such as computerized tomography, magnetic resonance imaging and positron emission tomography/computed tomography are commonly used for preoperative assessment of lymph node status. However, the measurement of lymph node size by these imaging techniques does not appear to be reliable indicator of LNM due to its limited accuracy [6, 7]. Meanwhile, current serum diagnostic biomarkers for GC, including carbohydrate antigen 724 (CA-724) and carcinoembryonic antigen (CEA) have shown low specificity and sensitivity for LNM prediction. Therefore, novel noninvasive biomarkers are urgently needed to complete and improve on current strategies for LNM prediction before surgery in GC.

MicroRNAs (miRNAs) are a subset of small non-coding RNAs (19–25 nucleotides in length) that regulate gene expression at post-transcriptional level by binding to the 3′ un-translated region (UTR) of target mRNAs [8]. Altered expression of miRNAs has been shown to be involved in regulation of crucial pathological processes in tumorigenesis, progression and metastasis [9, 10]. Accumulating evidence based on our and other published studies indicate that numerous stable miRNAs exist in human serum and have potential roles in diagnosis [11], histological classification [12] and prognosis assessment [13] in cancer. Recently, distinctive patterns of circulating miRNAs have been reported to predict lymph node involvement in various types of cancers, such as cholangiocarcinoma [14], colorectal cancer [15], and cervical squamous cell carcinoma [16]. In fact, differential expression of several circulating miRNAs including miR-1207-5p, miR-146a, and miR-148a have been reported in GC patients with LNM [17, 18]. However, these studies were limited by one or more of the following factors: limited number of screened miRNAs, small sample size, lack of independent validation and failure to identify unique serum miRNA signatures that could predict LNM in GC.

In the present study, we investigated serum miRNA expression profiles by miSeq sequencing followed by independent validations in a large cohort, with the intention to identify a panel of circulating miRNAs for prediction of lymph node status in GC. In addition, correlation between serum miRNAs and prognosis of GC patients with LNM was further assessed.

## RESULTS

### Identification of candidate miRNAs for LNM prediction by genome-wide miRNAs expression profiling

MiSeq sequencing technology was used to identify miRNAs with significantly altered expression among controls, GC patients with lymph node metastasis negative (LNNs) and GC patients with lymph node metastasis positive (LNPs). Based on the miSeq data, a total of 463 serum miRNAs were scanned, and among them 181, 225 and 158 miRNAs were detected in control group, LNNs group and LNPs group, respectively. Expression of a miRNA was considered “significantly altered” only if at least 20 copies were detected by miSeq sequencing, together with a larger than two-fold change in its expression level in LNPs vs. LNNs, LNPs vs. controls and LNNs vs. controls. Based on these criteria, 19 miRNAs were identified to be differentially expressed in LNPs and were thus further selected for RT-qPCR analysis (Supplementary Table 1).

### Evaluation of serum miRNAs by RT-qPCR analysis in training cohort

**Figure 1:**
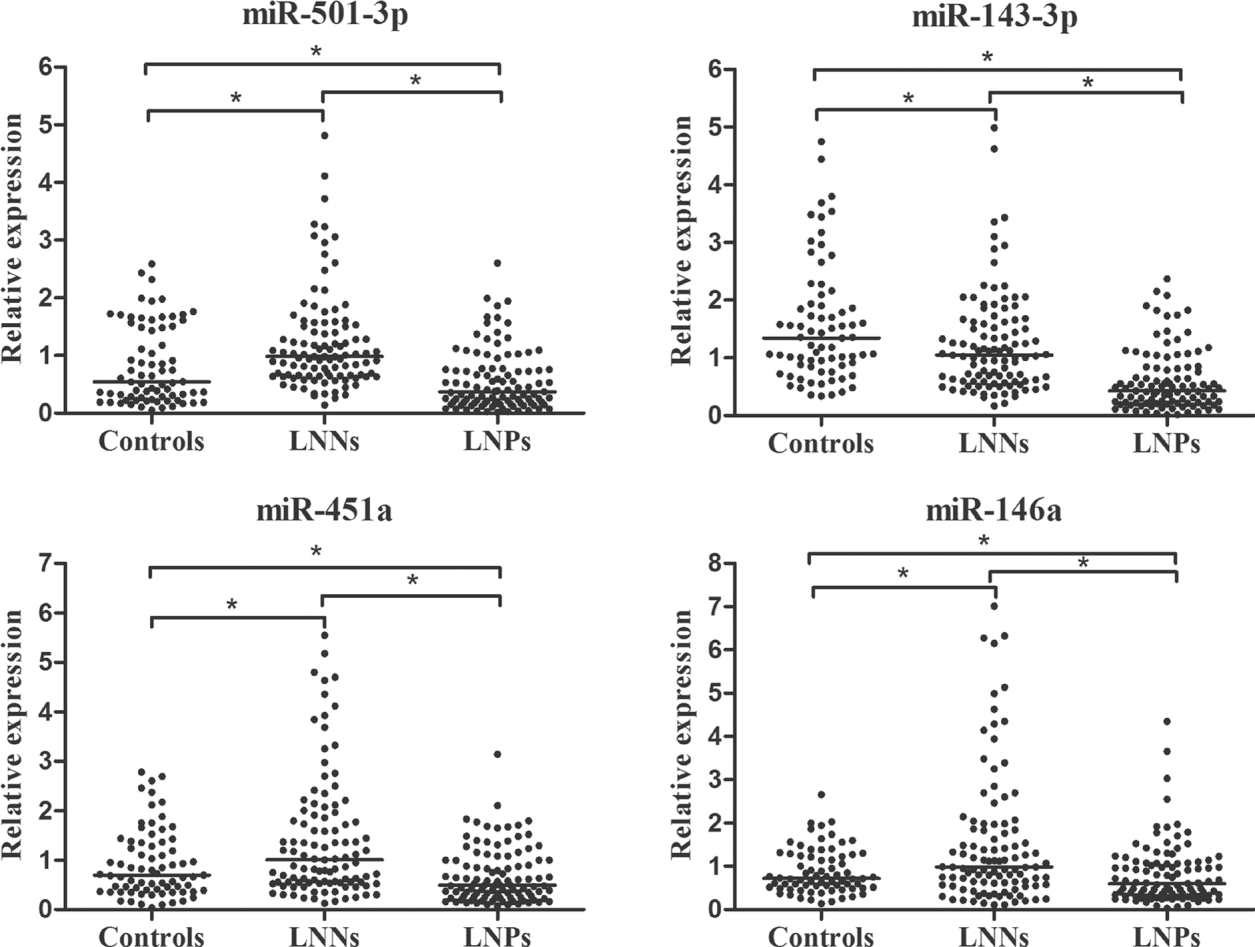
Relative expression of four selected serum miRNAs in controls (*n* = 73), LNNs (*n* = 103) and LNPs (*n* = 103) using RT-qPCR assay in training set, *p < 0.001

**Figure 2:**
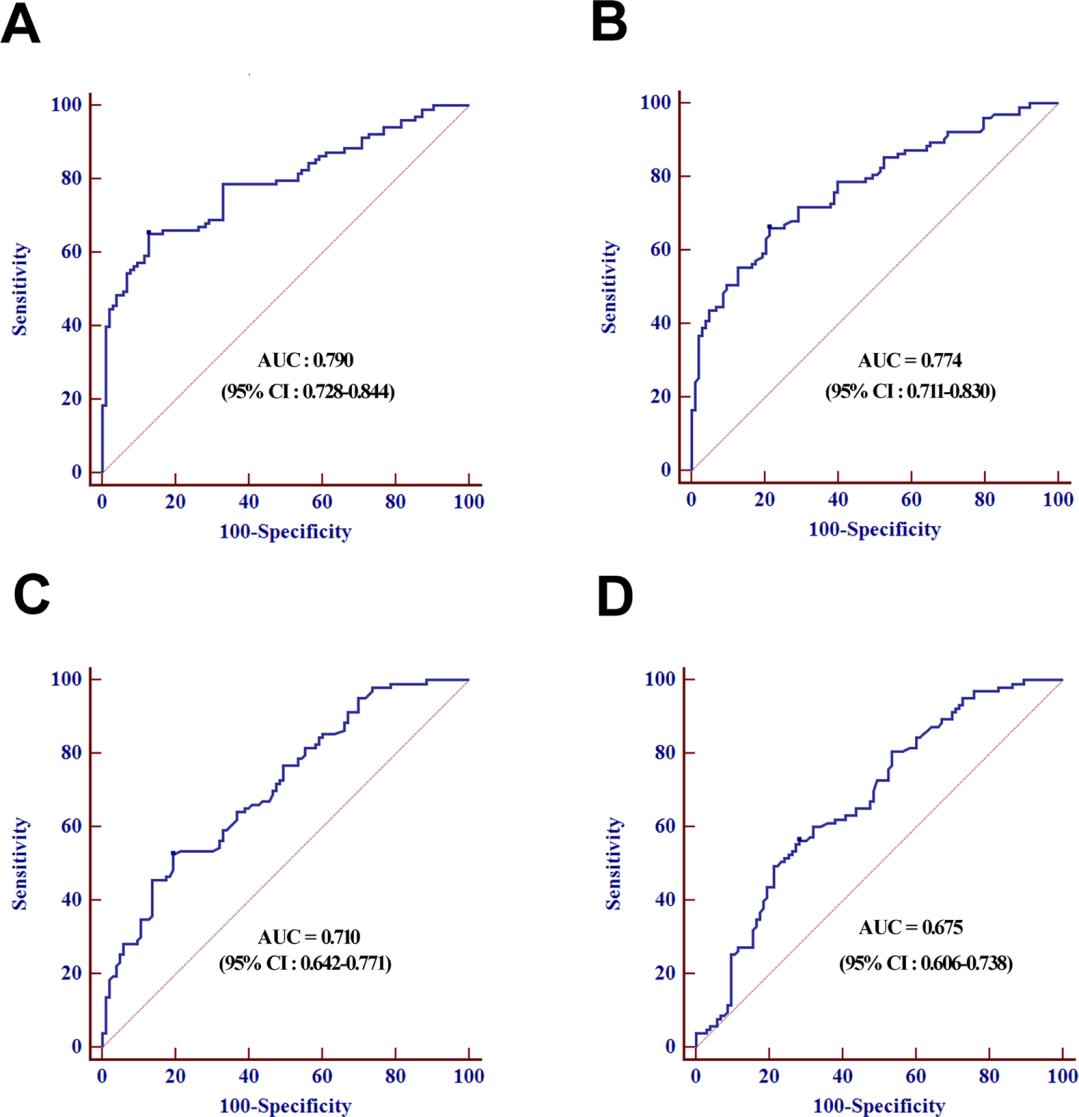
ROC curves analysis for the prediction of LNM using miR-501-3p (**A**), miR-143-3p (**B**), miR-451a (*C*), miR-146a (**D**) in training set.

The 19 differentially expressed candidate miRNAs were firstly tested with RT-qPCR using an independent cohort of 30 controls, 40 LNNs and 40 LNPs. Among these, 11 miRNAs passed the quality control (Supplementary Table 2). Four miRNAs (miR-501-3p, miR-143-3p, miR-451a, and miR-146a) among the 11 miRNAs showed differential expression levels among the three groups (all at *p* < 0.05, Supplementary Figure 1). The expression profile of these four miRNAs was further evaluated by RT-qPCR using additional 43 controls, 63 LNNs and 63 LNPs. These combined 73 controls, 103 LNNs and 103 LNPs was used as the training data set and four miRNAs showed differently expression among the three groups (all at *p* < 0.05, Figure 1). The predictive accuracy of miR-501-3p, miR-143-3p, miR-451a, and miR-146a for LNM measured by AUC was 0.790, 0.774, 0.710, and 0.675, respectively (Figure 2A–2D).

### Confirmation of serum miRNA concentrations in validation cohort

**Figure 3:**
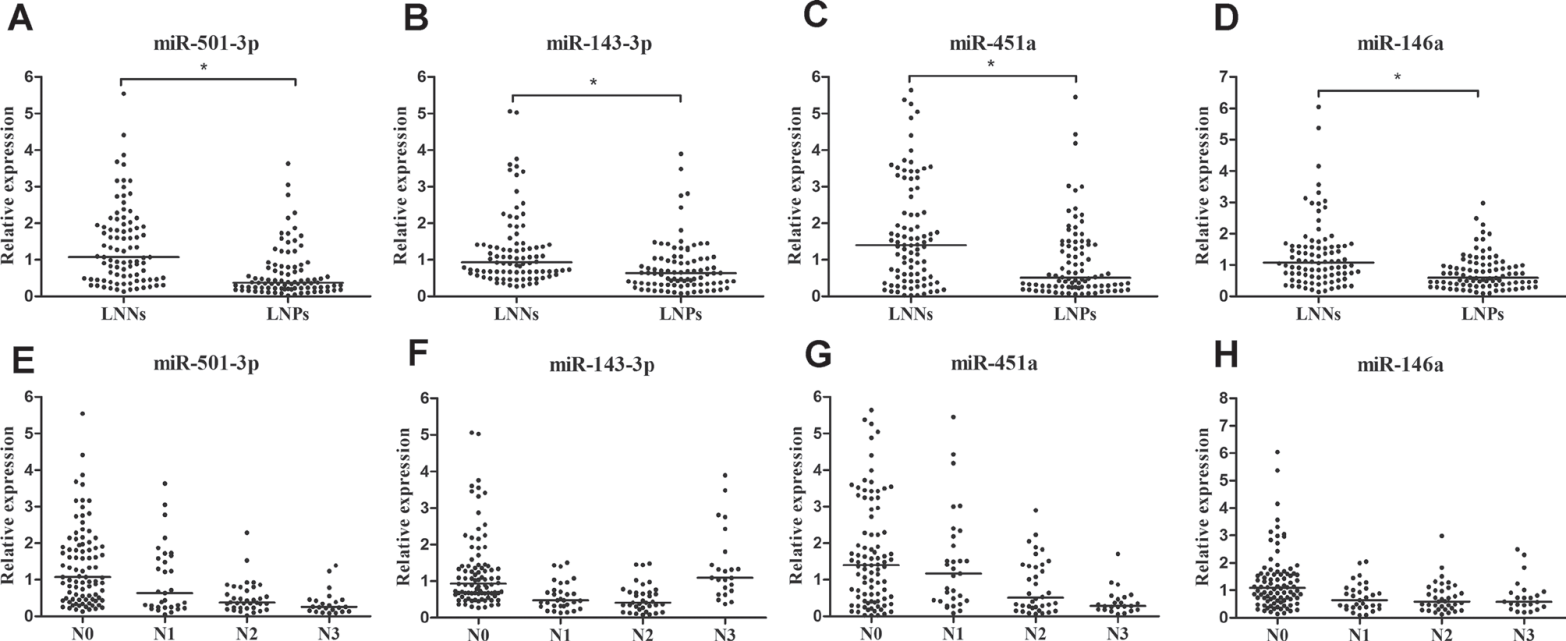
Relative expression of serum miR-501-3p, miR-451a, miR-143-3p and miR-146a in validation set (**A**–**D**) Concentrations of four miRNAs between LNNs (*n =* 90) and LNPs (*n =* 90), (**E**–**H**) concentrations of four miRNAs by different nodal stage.

The concentrations of these four identified miRNAs were further measured using a validation cohort consisting of 90 LNNs and 90 LNPs by RT-qPCR analysis. Alterations in the expression pattern of four miRNAs in validation set were consistent with those in training set (Table 1 and Figure 3A–3D). Expression of miR-501-3p, miR-143-3p, miR-451a, and miR-146a in serum samples from GC patients were significantly different by nodal stage (all at *p* < 0.05, Figure 3E–3H). The concentrations of miR-501-3p and miR-451a showed a tendency to decrease as nodal stage increased. The data presented in Supplementary Table 3 showed the relationship between the four miRNAs and clinicopathological characteristics of participants in validation set. Expression levels of miR-146a were shown to be correlated with depth of tumor invasion (*p* < 0.05). Moreover, lower levels of miR-501-3p, miR-143-3p, miR-451a, and miR-146a correlated with advanced clinical stage (all at *p* < 0.05). However, miRNA concentration did not differ by age, gender, tumor size, or cell differentiation (all at *p* > 0.05).

**Table 1:** Relative expression of four miRNAs in serum in controls, LNNs and LNPs in training set and validation set [median (interquartile range)]

	Training set			Validation set	
miRNA	Controls	LNNs	LNPs	LNNs	LNPs
miR-501-3p	0.54 (0.25–1.50)	0.98 (0.65–1.51)	0.37 (0.17–0.76)	1.07 (0.47–1.95)	0.37 (0.22–0.80)
miR-143-3p	1.34 (0.86–1.92)	1.05 (0.59–1.62)	0.43 (0.21–0.82)	0.93 (0.66–1.42)	0.63 (0.34–1.04)
miR-4 51a	0.70 (0.38–1.36)	1.01 (0.55–1.91)	0.49 (0.24–1.00)	1.40 (0.47–2.78)	0.51 (0.26–1.41)
miR-146a	0.72 (0.51–1.26)	0.98 (0.57–1.87)	0.60 (0.31–1.08)	1.08 (0.63–1.60)	0.59 (0.37–1.01)

### Establishing the predictive miRNA panel for LNM in GC using training cohort

**Figure 4:**
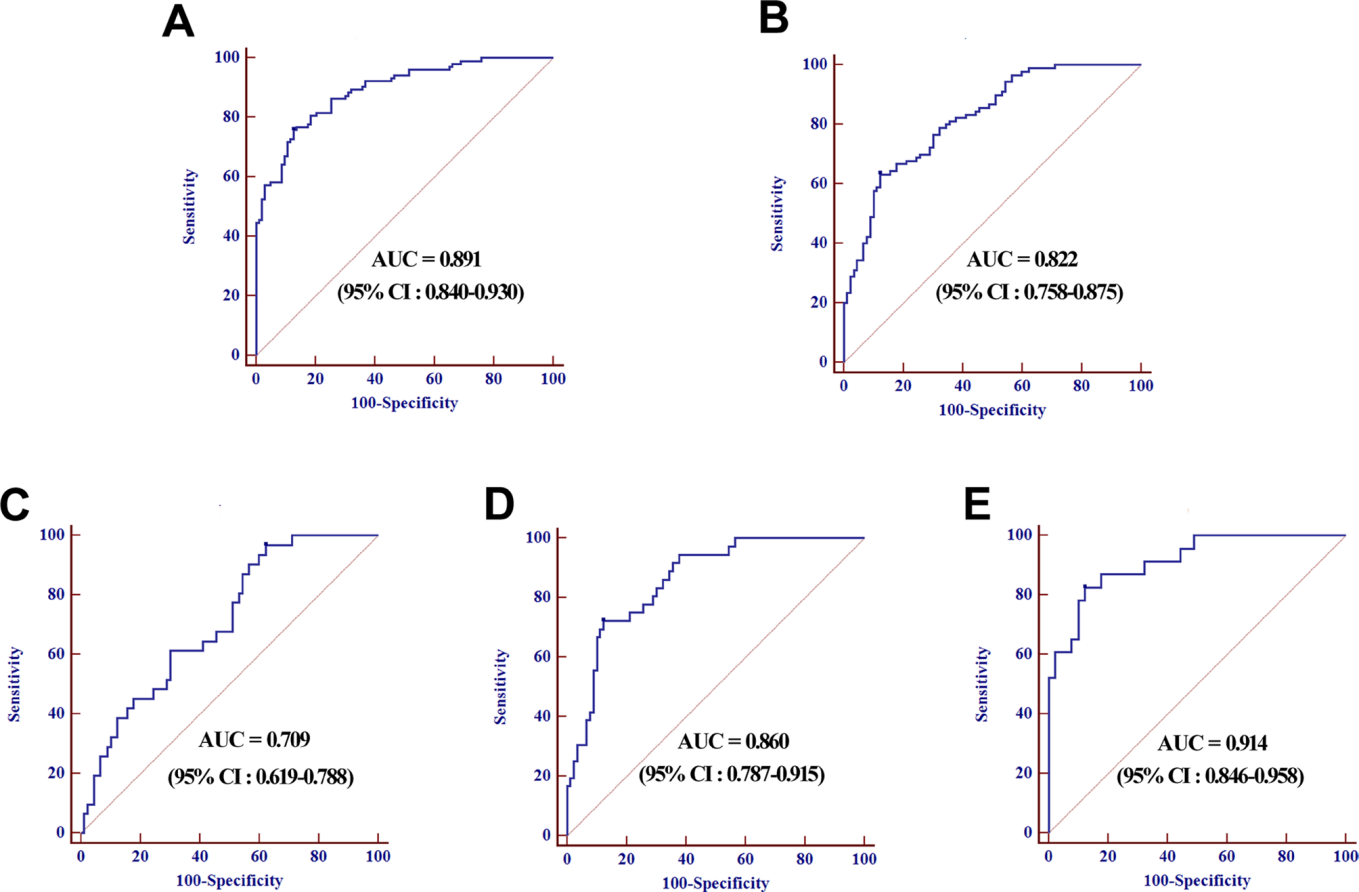
ROC curves analysis for the prediction of LNM using four-miRNA panel in training set (**A**) and validation set (**B**), ROC curves analysis using four-miRNA panel for the prediction of N1 (**C**), N2 (**D**), and N3 (**E**) in validation set.

Next, a stepwise logistic regression model to estimate the risk of being predicted with LNM was applied on training data set. The predicted probability of being predicted with LNM from the logit model based on the four-miRNA panel, logit (*p* = LNM) = –1.2020 + (0.3963 × miR-501-3p) + (0.3768 × miR-143-3p) + (0.2494 × miR-451a) + (0.2106 × miR-146a) was used to construct the ROC curve. The observed AUC of the four-miRNA panel was 0.891 (95% confidence interval [CI], 0.840 to 0.930) and the optimal cut-off value was –0.2398, providing a sensitivity of 75.73% and a specificity of 87.38% (Figure 4A). For a good predictive ability for LNM, a threshold of –0.2398 was selected.

### Validation of the predictive value of the miRNA panel using validation cohort

The parameters estimated from the training set were further used in a blind fashion to predict the probability of being diagnosed with LNM for the independent validation data set. Based on the classification threshold score of –0.2398 derived above, 116 samples were identified as LNNs and 64 samples were identified as LNPs. Subsequent unblinding of the results showed that 79 out of the 90 LNNs [specificity, 87.78% (95% CI, 79.2 to 93.7)] and 53 of the 90 LNPs [sensitivity, 63.33% (95% CI, 52.5 to 73.2)] were correctly identified, resulting in an AUC of 0.822 (95% CI, 0.758 to 0.875, Figure 4B).

In addition, ROC analysis was performed on the predictive value of the miRNA signature for LNPs with different lymph node status in the validation set. The AUCs of the panel for LNPs with N1, N2 and N3 were 0.709 (95% CI, 0.619 to 0.788), 0.860 (95% CI, 0.787 to 0.915) and 0.914 (95% CI, 0.846 to 0.958) respectively (Figure 4C–4E). Analysis of these classification results demonstrated that accuracy of the predictive miRNA signature for LNM trended upwards the higher lymph node status.

### Correlation of miRNA expression levels with OS in GC patients with LNM

**Figure 5:**
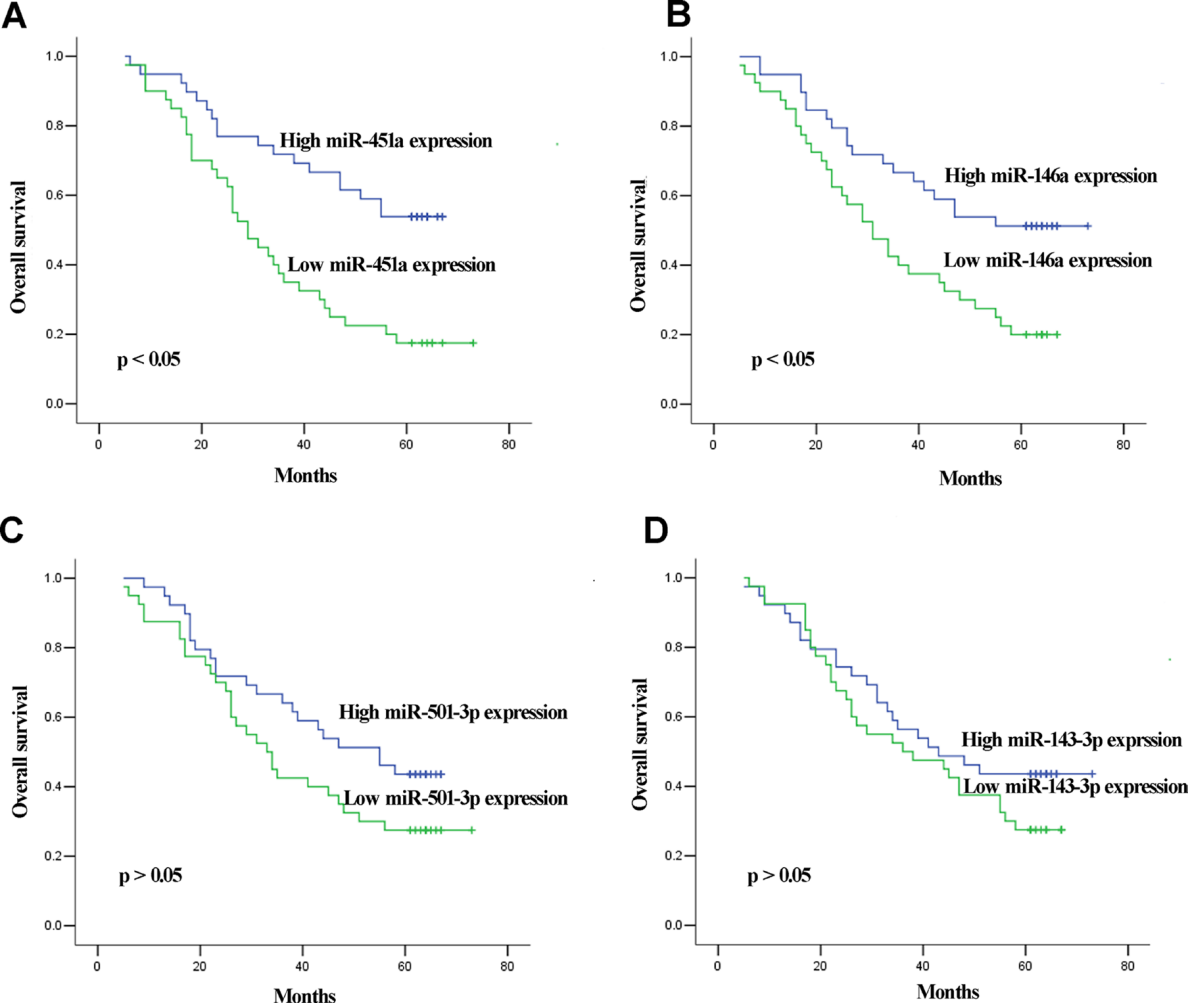
Kaplan–Meier curves for OS according to serum levels of miR-451a (**A**) and miR-146a (**B**), miR-501-3p (**C**), and miR-143-3p (**D**) in LNPs in validation set.

In the validation phase, 11 of the 90 LNPs were lost to follow-up and survival analysis was performed on the remaining 79 LNPs. The median follow-up time for OS information was 41 (range 5–73) months. The median of miRNAs expression was set as cutoff value to categorize LNPs into high or low level group. Kaplan–Meier survival analysis revealed that LNPs with lower miR-451a and miR-146a expression levels showed significantly reduced OS than those with high miR-451a and miR-146a expression levels (both at *p* < 0.05, Figure 5). In addition, we also performed univariate and multivariate analysis based on Cox proportional hazards regression model to explore factors associated with patient prognosis. The univariate analysis revealed that expression of miR-451a and miR-146a along with tumor stage were significantly correlated with OS of LNPs (*p* = 0.001, *p* = 0.016, and *p* < 0.001, respectively). The multivariate analysis showed that miR-451a expression (*p* = 0.028) and tumor stage (*p* = 0.011) maintained their significance as independent prognostic factors for OS of LNPs (Table 2).

**Table 2:** Univariate and multivariate Cox proportional hazards regression model analysis of OS in LNPs in validation set

Parameters	Categories	Univariate analysis		Multivariate analysis	
		HR (95% CI)	*p*	HR (95% CI)	*p*
Age	< 65 vs.≥ 65	0.775 (0.447–1.344)	0.365	0.624 (0.334–1.166)	0.139
Sex	Male vs. female	1.298 (0.747–2.254)	0.355	1.104 (0.569–2.143)	0.770
Tumor size	< 5 cm vs. ≥ 5 cm	1.013 (0.582–1.765)	0.962	1.208 (0.660–2.213)	0.540
Tumor stage	T1 vs.T2 vs.T3 vs.T4	1.878 (1.388–2.543)	< 0.001	1.681 (1.126–2.509)	0.011
Differentiation	Well vs. moderate vs. poor	0.914 (0.613–1.362)	0.657	0.861 (0.533–1.393)	0.542
miR-501-3p	Low vs. high	1.579 (0.906–2.754)	0.107	1.438 (0.764–2.710)	0.261
miR-143–3p	Low vs. high	1.398 (0.803–2.435)	0.237	1.007 (0.531–1.910)	0.984
miR-451a	Low vs. high	2.710 (1.514–4.849)	0.001	1.972 (1.704–3.622)	0.028
miR-146a	Low vs. high	0.503 (0.287–0.880)	0.016	1.624 (0.871–3.028)	0.127

## DISCUSSION

Most of studies mainly focused on miRNA expression in GC tumor tissues and cell lines. Chang et al. demonstrated that overexpression of miR-125b could promote cell migration and invasion in GC tissues by targeting STARD13 and NEU1 [19]. Chen and colleagues revealed that aberrant expression of miR-10a in tissues coul s for GC metastasis prediction, the procedure of collecting tissues samples is invasive and depends on surgical resection. Circulating miRNAs have emerged as potential novel noninvasive biomarkers for predicting lymph node status of cancer [14–16]. Here, we demonstrated that four miRNAs (miR-501-3p, miR-143-3p, miR-451a, and miR-146a) were differently expressed in a LNM-specific manner. Our results highlighted that serum miRNA signature based on these four miRNAs could have the potential to be used as new biomarkers for LNM prediction in GC with high accuracy. Moreover, miR-451a was also identified as an independent factor for prognosis of LNM patients. This is the first study to establish a model using circulating miRNAs to predict lymph node status in GC via the high-throughput platform.

Several studies have investigated serum miRNA profiles as fingerprints for diagnosis of GC [20, 21]. Yet, significant efforts to identify serum miRNA signatures for LNM prediction in GC have met with limited success. Consistent with our findings, down-regulation of miR-146a in serum of LNPs has already been reported by Kim et al. [17]. They also demonstrated that serum concentrations of miR-21 and miR-148a were associated with pN stage of GC. Our study confirmed the expression of miR-148a among 10 controls, 10 LNNs and 10 LNPs with the deep sequencing platform. However, we did not further evaluate it by RT-qPCR, because it failed to pass our selection criteria. In addition, Imaoka and colleagues revealed that expression of miR-203 was significantly lower in LNPs compared with LNNs [22]. Most of these studies assessed the serum levels of miRNAs that were selected on the basis of miRNA data from cancer tissues. Nevertheless, the expression patterns of serum miRNAs would not be identical to those in cancer cells and tissues as circulating miRNAs might not only be derived from tumor-cell lysis but can also be actively secreted from miRNA-protein complexes [23] and/or cell-derived microvesicles [24]. In comparison, we performed genome-wide serum miRNAs analysis via miSeq sequencing technology. Considering individual variation in miSeq information from pooled samples, candidate miRNAs revealed by sequencing were validated by two phases of RT-qPCR assays using a large cohort. Finally, a four-miRNA signature with high accuracy for LNM prediction in GC was constructed and subsequently validated by two phases of RT-qPCR analysis using different cohorts.

Understanding the targets and the molecular mechanisms by which the miRNAs regulate GC development at tissue level, could help to promote their clinical application. Wu et al. demonstrated that miR-143 could suppress cell growth and induce apoptosis by targeting COX-2 in GC [25]. Expression level of miR-143 was also observed to be decreased in GC tissues and combined transfection of miR-143 and miR-145 in GC cells resulted in additive growth inhibition [26]. Moreover, overexpression of miR-451 in GC cells could reduce cell proliferation and increase sensitivity to radiotherapy by targeting oncogene macrophage migration inhibitory factor [27]. In addition, Li et al. showed that down-regulation of miR-146a-5p in tissues correlated with more extensive lymph node metastasis and lymphatic invasion in GC [28]. Although miR-501 has been shown to promote cell proliferation in hepatocellular carcinoma [29], our study is the first to report the importance of miR-501 expression profile in association with lymph node status in GC. Additional researches studies are required to improve our understanding about the regulatory mechanisms of these miRNAs, along with their roles in molecular pathogenesis.

The dysregulation of miRNA expression in GC tissues has been shown to be correlated with patient survival [30, 31]. This prompted us to analyze the correlation between circulating miRNAs and prognosis of LNM patients in GC. In this study, we further analyzed correlation between four miRNAs and survival of LNM patient. Kaplan–Meier survival analysis revealed that low miR-451a expression level correlated with shorter OS of LNPs. Taking a step further, Cox proportional hazards regression model analysis displayed that serum miR-451a level was independent factor influencing OS of LNPs. Several other investigators have reported similar findings in GC tissues. Brenner and colleagues identified miR-451 in tissues as a potential prognostic factor for GC [32]. Su et al. demonstrated that down-regulation of miR-451 in GC tissues showed positive correlation with lymphatic metastasis, clinical staging and shorter overall survival of patients [33]. Thus based on our and previously reported findings, we speculated that pretreatment serum levels of miR-451a might also serve as new prognostic biomarkers for LNM patients.

Prediction of LNM in GC before surgery noninvasively could help to guide on the need for surgical lymph node resection. For early LNPs, less invasive treatment such as endoscopic mucosal resection can be immediately conducted without delays and thus can be effective. Endoscopic resection of tumor should be avoided when there is risk for LNM, even in pT1 patients [34, 35]. For localized LNNs, limited lymph node dissection by surgery is recommended to reduce postoperative mortality. If GC patients were diagnosed at advanced stage with LNM, surgical resection with extensive lymphadenectomy is necessary for better outcome [36]. Here, a four miRNA panel in serum might provide relatively definitive answer as to lymph node status, thus enabling a defined treatment pathway. Nevertheless, this study included only 203 LNNs and 203 LNPs from a single institution. Also, it is not clear whether the predictive miRNA panel is capable of discriminating LNPs from other types of invasive tumors. As the clinical utility of these putative serum biomarkers to accurately predict patients with LNM is ultimate goal, this work should be viewed as an important first step but not the definitive answer.

In conclusion, we defined a distinctive serum miRNA signature for LNM prediction in GC and also identified independent prognostic variables for LNPs. These findings may provide a foundation for the development of novel noninvasive test to predict lymph node involvement and determination of innovative therapeutic strategies.

## MATERIALS AND METHODS

### Study design, patients and control subjects

**Figure 6:**
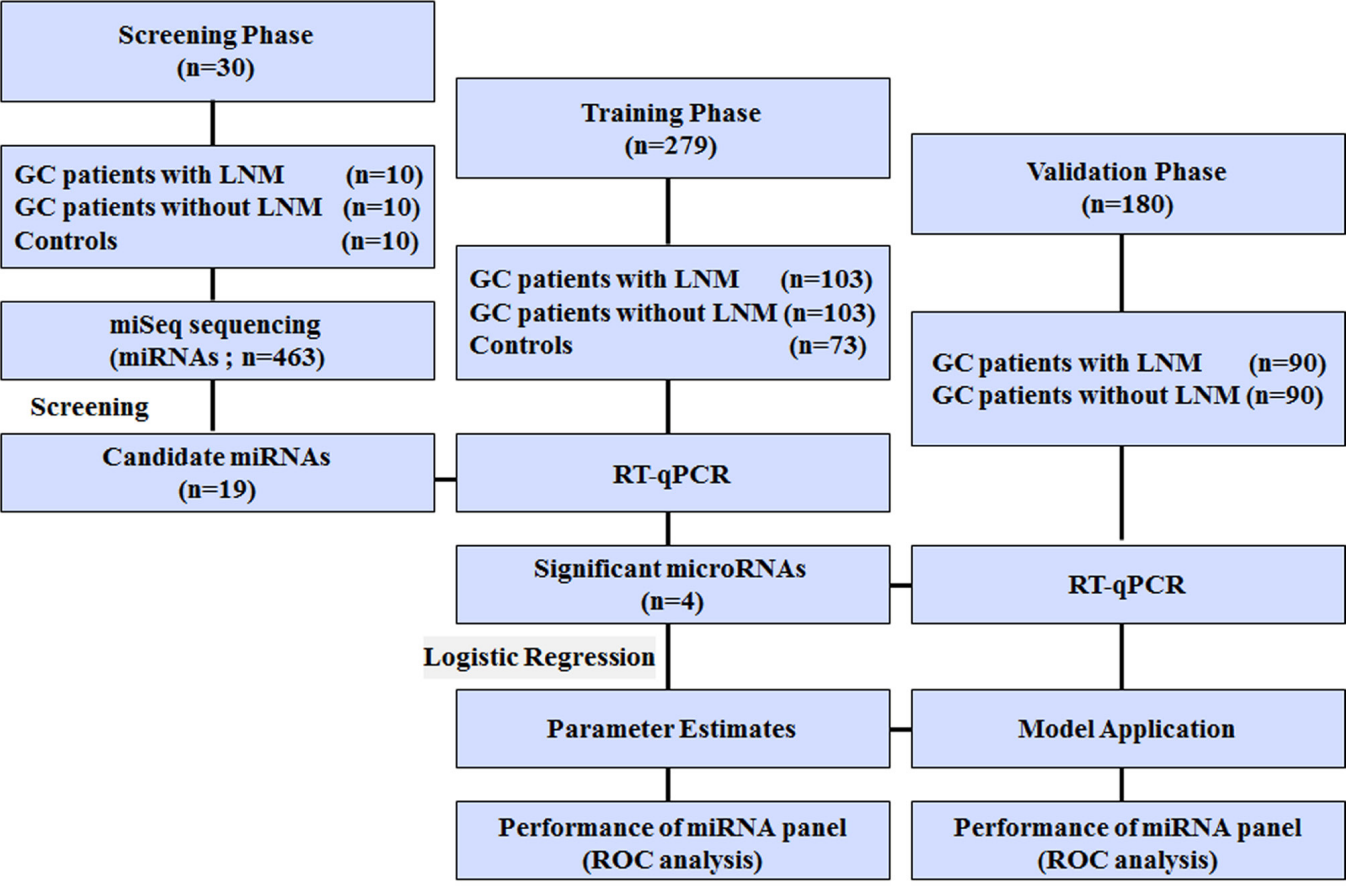
Study outline

This study included 203 LNPs, 203 LNNs and 83 healthy control individuals that were recruited from Qilu Hospital, Shandong University, between January 2007 and February 2011. All participants were randomly allocated to three phases (Figure 6). For initial biomarker screening phase, pooled serum samples from 10 controls, 10 LNNs and 10 LNPs were subjected to miSeq sequencing technology and miRNAs with significant differences in expression levels among three groups were identified. In the training phase, candidate miRNAs were firstly verified by reverse transcription quantitative real-time PCR (RT-qPCR) in serum samples from 30 controls, 40 LNNs and 40 LNPs. Subsequently, miRNAs differentially expressed were further analyzed in additional 43 controls, 63 LNNs and 63 LNPs. The overall data from these 103 LNNs and 103 LNPs were used to construct the predictive miRNA panel for LNM prediction in GC based on logistic regression model. In the validation phase, serum samples from another cohort of 90 LNNs and 90 LNPs were prospectively entered into the discriminatory model to validate the predictive accuracy of the constructed algorithm. Additionally, these LNPs were followed up at intervals of 3 months during the first 2 years and 6 months up to the fifth year. The date of the latest retrieved record was March 31, 2016.

Histopathology of all LNNs and LNPs was confirmed by surgical resection of tumors. Tumors were staged according to the tumor-node-metastasis staging system of the International Union against Cancer, and histological grade was assessed according to the World Health Organization (WHO) criteria. Control subjects were recruited from a large pool of individuals seeking a routine health check-up at the Healthy Physical Examination Centre of Qilu Hospital, Shandong University. People who showed no evidence of disease and without a history of GC were selected as tumor-free controls. Demographic and clinical features of the participants are summarized in Supplementary Table 4. No significant differences were observed among LNPs, LNNs and control subjects in distribution of smoking, alcohol consumption, age and gender. This study was approved by the Clinical Research Ethics Committee of Qilu Hospital, Shandong University and informed consent was obtained from each participant.

### Preparation of serum samples

Blood samples were collected prior to any therapeutic procedures at the day before tumor resection. The 5ml venous blood from each participant was centrifuged at 4000 rpm for 10 min at 4°C within 2 h of collection, followed by a second centrifugation at 12000 rpm for 15 min at 4°C to eliminate any residual cells debris. Supernatant serum was then stored at –80°C until further processing.

### miSeq sequencing

For miSeq sequencing, total RNA of each group of serum samples was used to prepare the miRNA sequencing library with NEBNext^®^Multiplex Small RNA Library Prep Set for Illumina^®^ (NEB Co., USA). After quantification on an Agilent 2100 Bioanalyzer, the library was denatured with 0.1M NaOH to generate single-stranded DNA molecules, which were captured on flow cells, amplified *in situ* and finally sequenced on miSeq following manufacturer’s (Illumina) protocol. Off-line basecaller software (OLB V1.8.0) was used for image analysis and base calling. Then, index sequences were trimmed from clean reads (reads that passed Solexa CHASTITY quality filter) and the reads no longer than 8 nt were excluded. Later, reads passing the filter (length longer than 15 nt) were mapped to the latest human reference miRNA precursor set (Sanger miRBase 17.0) using the Novoalign software (v2.07.11).

### Quantification of serum miRNAs by RT-qPCR analysis

RT-qPCR was conducted by ABI PRISM 7500 Sequence Detection System (Applied Biosystems, Foster City, CA, USA) using the SYBR PrimeScript miRNA RT-qPCR Kit (Takara Bio Inc). Firstly, the 2 × preparation buffer consisting of 2.5% Tween 20 (EMD Chemicals, Gibbstown, NJ, USA), 50 mmol/L Tris (Sigma-Aldrich, St.Louis, MO, USA), and 1 mmol/L EDTA (Sigma-Aldrich, St.Louis, MO, USA) was prepared [37]. Then, the 20 μl reverse transcription (RT) reaction system consisted of 3 μl of serum that mixed with 3 μl of 2× preparation buffer, 10 μl of 2 × miRNA Reaction Buffer Mix, 2 μl of miRNA Primescript RT Enzyme Mix, and 2 μl of 0.1% BSA. The RT reactions were performed on the following conditions: 37°C for 60 min and 85°C for 5s followed by 4°C for 60 min. The generated cDNAs were centrifuged at 16,000 g for 10 min at 4°C and was diluted by 5-fold. The 25 μl RT-qPCR reaction system contained 12.5 μl of SYBR Premix Ex Taq II, 0.5 μl of Dye II, 2 μl of 5 μM of forward primer, 1 μl of 10 μM of Uni-miR RT-qPCR Primer, 7 μl of ddH_2_O and 2 μl of template cDNA. The amplification was carried out as follows; 95°C for 30s, followed by 45 cycles of 95°C for 5s and 57°C for 34s. All reactions were performed in triplicate and Ct values were measured with default threshold settings. Specificity of the RT-qPCR product was confirmed using melting curve analysis, and miRNAs with a Ct value of more than 35 and a detection rate of less than 75% in each group were excluded from further analyses. U6 was used as reference gene and the relative expression levels of target miRNAs was calculated by using the 2^–∆∆Ct^ method [38].

### Statistical analysis

Normality of the distribution of data in each group was determined by Kolmogorov-Smirnov test. Non-parametric Mann-Whitney *U* tests were employed to compare differences in expression levels of serum miRNAs between two groups and Kruskal-Wall tests were used for comparison among more than three groups. Receiver operating characteristic (ROC) curves were established to discriminate LNPs from LNNs. Area under the ROC curve (AUC) was used as an accuracy index for evaluating the predictive performance of constructed miRNA panel. The Youden index (sensitivity + specificity - 1) was used to set the optimal cutoff point [39]. Survival curves were estimated with Kaplan–Meier method and log-rank test was used to compare the distributions of survival times. Cox proportional hazards regression model was employed to evaluate independent factors of overall survival (OS). Matlab software (Matlab, R2013a) was used for logistic regression analysis, MedCalc 9.3.9.0 (MedCalc, Mariakerke, Belgium) was employed for ROC analysis, and SPSS version 17.0 software (SPSS Inc., Chicago, IL, USA) was used for other analysis. Statistical significance was defined as a two-sided *p* value of less than 0.05.

## SUPPLEMENTARY MATERIALS FIGURE AND TABLES



## Abbreviations

ROC: receiver operating characteristic
AUC: area under the ROC curve
miRNA: microRNA
RT-qPCR: reverse transcription quantitative real-time PCR
CI: confidence interval
GC: gastric cancer
LNM: lymph node metastasis
LNP: lymph node metastasis positive
LNN: lymph node metastasis negative
