# Supplementary material for: Identification of circulating microRNA signatures as potential noninvasive biomarkers for prediction and prognosis of lymph node metastasis in gastric cancer
Source: Oncotarget. 2017 May 10;8(39):65132–42. doi: 10.18632/oncotarget.17789 (PMC5630318; doi:10.18632/oncotarget.17789)
Supplement: Supplementary file 1 [file oncotarget-08-65132-s001.pdf]

# Identification of circulating microRNA signatures as potential noninvasive biomarkers for prediction and prognosis of lymph node metastasis in gastric cancer

## SUPPLEMENTARY MATERIALS

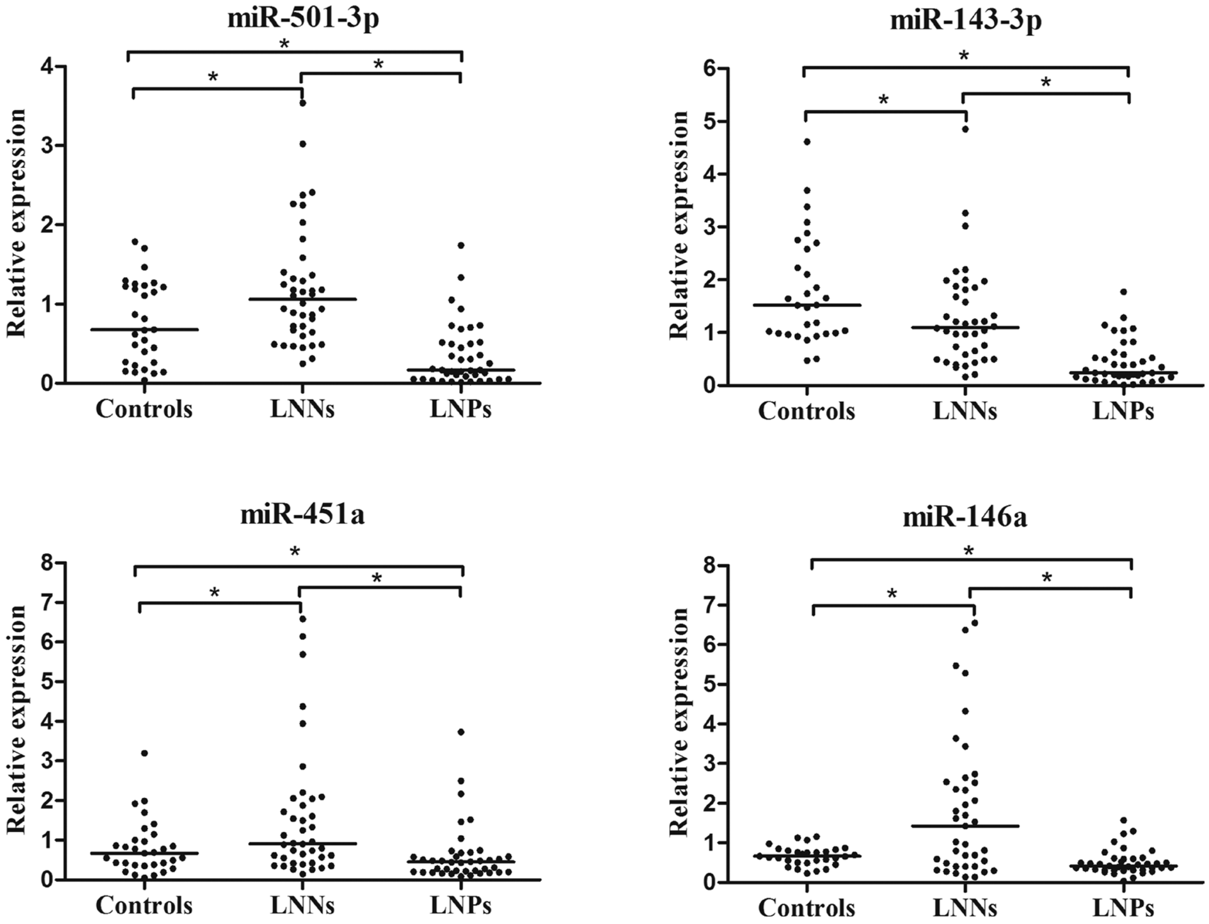


**Supplementary Figure 1: Relative expression of four selected serum miRNAs in Controls ($n$ = 30), LNNs ($n$ = 40) and LNPs ($n$ = 40) using RT-qPCR assay in training set, *$p < 0.05$.**

**Supplementary Table 1: Altered serum miRNAs in Controls, LNNs and LNPs determined by miSeq sequencing**

| Dysregulated miRNA | Controls | LNNs | LNPs | LNNs/Controls | LNPs/Controls | LNPs/LNNs |
|---|---|---|---|---|---|---|
| miR-10a-5p | 611 | 277 | 119 | 0.45 | 0.19 | 0.43 |
| miR-126-5p | 1820 | 80 | 594 | 0.04 | 0.33 | 7.43 |
| miR-16-5p | 28565 | 3411 | 10765 | 0.12 | 0.38 | 3.16 |
| miR-27a-3p | 208 | 20 | 77 | 0.10 | 0.37 | 3.85 |
| miR-451a | 1870 | 179 | 916 | 0.10 | 0.49 | 5.12 |
| miR-486-3p | 48 | 21 | 157 | 0.44 | 3.27 | 7.48 |
| miR-501-3p | 287 | 142 | 65 | 0.49 | 0.23 | 0.46 |
| miR-143-3p | 4891 | 2202 | 958 | 0.45 | 0.19 | 0.44 |
| miR-146a | 252 | 2237 | 526 | 8.88 | 2.09 | 0.24 |
| miR-98-5p | 112 | 772 | 306 | 6.89 | 2.73 | 0.40 |
| miR-432-5p | 33 | 306 | 76 | 9.27 | 2.30 | 0.25 |
| miR-4446-3p | 118 | 47 | 22 | 0.40 | 0.19 | 0.47 |
| miR-151a-3p | 41 | 382 | 135 | 9.32 | 3.29 | 0.35 |
| let-7i-5p | 349 | 2414 | 943 | 6.92 | 2.70 | 0.39 |
| miR-320a | 3218 | 20305 | 9720 | 6.30 | 3.02 | 0.48 |
| miR-378a-3p | 52 | 518 | 206 | 9.96 | 3.96 | 0.40 |
| miR-382-5p | 33 | 263 | 70 | 7.97 | 2.12 | 0.27 |
| miR-93-5p | 120 | 1553 | 291 | 12.94 | 2.43 | 0.19 |
| miR-574-5p | 20 | 130 | 42 | 6.50 | 2.10 | 0.32 |

**Supplementary Table 2: Ct values of serum miRNAs in Controls, LNNs and LNPs in miSeq sequencing determined by RT-qPCR analysis**

| dysregulated miRNAs | Controls | | | LNNs | | | LNPs | | |
|---|---|---|---|---|---|---|---|---|---|
| | Ct Min | Ct Max | Mean ± SD | Ct Min | Ct Max | Mean ± SD | Ct Min | Ct Max | Mean ± SD |
| miR-10a-5p | 28.98 | 35.32 | 31.49 ± 1.60 | 27.95 | 34.98 | 31.37 ± 1.70 | 26.87 | 34.47 | 30.63 ± 2.11 |
| miR-126-5p | 33.08 | 37.98 | 35.39 ± 1.17 | 34.2 | 39.71 | 36.52 ± 1.24 | 34.12 | 38.22 | 36.12 ± 0.90 |
| miR-16-5p | 34.08 | 39.54 | 35.87 ± 1.42 | 31.12 | 40.19 | 35.34 ± 2.17 | 33.33 | 38.57 | 35.96 ± 1.24 |
| miR-27a-3p | 28.76 | 32.04 | 30.51 ± 0.83 | 26.38 | 32 | 29.64 ± 1.05 | 27.59 | 32.14 | 30.06 ± 1.05 |
| miR-451a | 30.78 | 35 | 32.68 ± 1.03 | 27.72 | 32.81 | 30.08 ± 1.18 | 28.75 | 34.17 | 31.10 ± 1.28 |
| miR-486-3p | 27.05 | 30.64 | 28.83 ± 1.03 | 26.72 | 30.95 | 28.85 ± 1.12 | 26.17 | 30.13 | 28.10 ± 1.09 |
| miR-501-3p | 28.76 | 34.79 | 31.87 ± 1.25 | 27 | 31.97 | 29.73 ± 0.98 | 29.9 | 34.85 | 32.23 ± 1.38 |
| miR-143-3p | 27.93 | 32.98 | 30.34 ± 1.39 | 27.87 | 32.87 | 29.91 ± 1.01 | 28.39 | 39.19 | 32.52 ± 1.99 |
| miR-146a | 31.07 | 34.33 | 33.23 ± 0.68 | 26.86 | 34.34 | 31.32 ± 1.50 | 30.8 | 36.72 | 32.99 ± 1.32 |
| miR-98-5p | 34.15 | 41.43 | 37.03 ± 1.74 | 33.91 | 43.22 | 36.47 ± 1.99 | 34.22 | 42.65 | 36.86 ± 1.78 |
| miR-432-5p | 34.55 | 41.34 | 38.24 ± 1.83 | 33.38 | 38.52 | 35.54 ± 1.11 | 34.45 | 41.45 | 37.34 ± 1.62 |
| miR-4446-3p | 25.66 | 30.55 | 27.96 ± 1.32 | 25.32 | 29.87 | 27.28 ± 1.15 | 24.91 | 29.55 | 26.84 ± 1.19 |
| miR-151a-3p | 27.54 | 31.22 | 29.57 ± 1.09 | 27.54 | 30.84 | 29.01 ± 0.89 | 27.45 | 34.04 | 29.97 ± 1.38 |
| let-7i-5p | 35.65 | 43.25 | 38.64 ± 1.77 | 36.09 | 40.37 | 37.92 ± 1.10 | 35.43 | 44.61 | 38.54 ± 1.71 |
| miR-320a | 33.09 | 37.89 | 35.78 ± 1.23 | 32.42 | 37.66 | 35.1 ± 1.17 | 33.02 | 41.01 | 35.81 ± 1.66 |
| miR-378a-3p | 35.03 | 42.07 | 37.94 ± 1.81 | 33.25 | 43.22 | 37.17 ± 2.18 | 33.52 | 40.54 | 37.29 ± 1.73 |
| miR-382-5p | 28.65 | 32.98 | 31.54 ± 1.04 | 28.98 | 32.98 | 31.30 ± 0.99 | 28.98 | 39.98 | 31.43 ± 1.75 |
| miR-93-5p | 33.65 | 39.76 | 36.74 ± 1.35 | 31.42 | 39.35 | 35.7 ± 1.73 | 34.12 | 40.97 | 36.46 ± 1.57 |
| miR-574-5p | 25.87 | 29.53 | 27.75 ± 0.95 | 26.06 | 30.32 | 28.01 ± 1.15 | 26.12 | 31.23 | 28.58 ± 1.46 |

**Supplementary Table 3: Relative expression of serum miRNAs in participants according to clinicopathological characteristics in validation set**

| Variables | *n* | miR-501-3p | | miR-143-3p | | miR-451a | | miR-146a | |
|---|---|---|---|---|---|---|---|---|---|
| | | median (interquartile range) | *p* | median (interquartile range) | *p* | median (interquartile range) | *p* | Median (interquartile range) | *p* |
| **Age** | | | | | | | | | |
| < 65 | 81 | 0.59 (0.23-1.55) | 0.29 | 0.85 (0.48–1.41) | 0.20 | 1.15 (0.27–1.79) | 0.60 | 0.91 (0.48–1.41) | 0.25 |
| ≥ 65 | 99 | 0.65 (0.34–1.72) | | 0.69 (0.44–1.26) | | 0.76 (0.33–1.89) | | 0.75 (0.44–1.29) | |
| **Gender** | | | | | | | | | |
| Male | 98 | 0.64 (0.31–1.56) | 0.90 | 0.69 (0.47–1.22) | 0.14 | 0.74 (0.33–1.71) | 0.76 | 0.84 (0.46–1.32) | 0.87 |
| Female | 82 | 0.59 (0.22–1.83) | | 1.04 (0.48–1.44) | | 1.10 (0.27–1.88) | | 0.83 (0.39–1.45) | |
| **Tumor size (cm)** | | | | | | | | | |
| < 5 | 103 | 0.70 (0.34–1.60) | 0.42 | 0.72 (0.41–1.40) | 0.75 | 0.64 (0.29–1.58) | 0.20 | 0.73 (0.42–1.33) | 0.23 |
| ≥ 5 | 77 | 0.56 (0.25–1.55) | | 0.69 (0.47–1.13) | | 1.35 (0.30–1.89) | | 0.93 (0.50–1.38) | |
| **Cell differentiation** | | | | | | | | | |
| Well | 70 | 0.65 (0.34–1.76) | 0.74 | 0.69 (0.37–1.42) | 0.60 | 0.65 (0.26–1.40) | 0.26 | 0.73 (0.39–1.25) | 0.07 |
| Moderate | 63 | 0.71 (0.26–1.34) | | 0.70 (0.45–1.09) | | 0.95 (0.35–1.59) | | 0.98 (0.48–1.53) | |
| Poor | 47 | 0.55 (0.23–1.59) | | 0.73 (0.57–1.41) | | 1.36 (0.29–2.23) | | 0.73 (0.43–1.14) | |
| **Depth of invasion** | | | | | | | | | |
| T1 | 33 | 0.82 (0.34–1.63) | 0.82 | 0.89 (0.60–1.45) | 0.23 | 1.58 (0.27–3.09) | 0.29 | 1.32 (0.74–1.64) | < 0.05 |
| T2 | 55 | 0.49 (0.25–1.60) | | 0.65 (0.41–1.28) | | 0.92 (0.27–1.71) | | 0.72 (0.32–1.05) | |
| T3 | 56 | 0.70 (0.29–1.83) | | 0.79 (0.46–1.29) | | 0.97 (0.36–1.87) | | 0.72 (0.43–1.22) | |
| T4 | 36 | 0.59 (0.25–1.91) | | 0.68 (0.39–1.08) | | 0.62 (0.27–1.41) | | 0.82 (0.46–1.49) | |
| **N category** | | | | | | | | | |
| N0 | 90 | 1.07 (0.47–1.95) | < 0.05 | 0.93 (0.66–1.42) | < 0.05 | 1.40 (0.47–2.77) | < 0.05 | 1.08 (0.63–1.60) | < 0.05 |
| N1 | 31 | 0.63 (0.25–1.66) | | 0.48 (0.31–0.75) | | 1.17 (0.43–2.18) | | 0.64 (0.37–1.06) | |
| N2 | 36 | 0.38 (0.22–0.77) | | 0.41 (0.23–0.69) | | 0.51 (0.23–1.40) | | 0.58 (0.31–0.99) | |
| N3 | 23 | 0.25 (0.13–0.45) | | 1.09 (0.73–1.80) | | 0.28 (0.18–0.52) | | 0.73 (0.39–1.21) | |
| **Stage** | | | | | | | | | |
| I | 66 | 0.93 (0.34–1.92) | < 0.05 | 1.00 (0.49–1.44) | < 0.05 | 0.99 (0.56–1.44) | < 0.05 | 1.28 (0.51–2.23) | < 0.05 |
| II | 55 | 0.49 (0.29–1.39) | | 0.70 (0.47–1.09) | | 0.79 (0.43–1.51) | | 0.92 (0.28–2.23) | |
| III | 59 | 0.47 (0.26–1.23) | | 0.67 (0.38–1.04) | | 0.61 (0.35–1.12) | | 0.49 (0.19–1.40) | |

## Supplementary Table 4: Clinical characteristics of participants included in the study

| Variables | Screening phase | | | Training phase | | | Validation phase | |
|---|---|---|---|---|---|---|---|---|
| | Controls | LNNs | LNPs | Controls | LNNs | LNPs | LNNs | LNPs |
| | (*n* =10) | (*n* =10) | (*n* = 10) | (*n* =73) | (*n* = 103) | (*n* =103) | (*n* = 90) | (*n* = 90) |
| **Age** | | | | | | | | |
| < 65 | 6 (60.0%) | 7 (70.0%) | 5 (50.0%) | 31 (42.5%) | 47 (45.6%) | 39 (37.9%) | 38 (42.2%) | 43 (47.8%) |
| ≥ 65 | 4 (40.0%) | 3 (30.0%) | 5 (50.0%) | 42 (57.5%) | 56 (54.4%) | 64 (62.1%) | 52 (57.8%) | 47 (52.2%) |
| **Gender** | | | | | | | | |
| Male | 5 (50.0%) | 6 (60.0%) | 7 (70.0%) | 46 (63.0%) | 59 (57.3%) | 64 (62.1%) | 47 (52.2%) | 51 (56.7%) |
| Female | 5 (50.0%) | 4 (40.0%) | 3 (30.0%) | 27 (37.0%) | 44 (42.7%) | 39 (37.9%) | 43 (47.8%) | 39 (43.3%) |
| **Smoking status** | | | | | | | | |
| Never | 6 (60.0%) | 8 (80.0%) | 8 (80.0%) | 43 (58.9%) | 72 (69.9%) | 58 (56.3%) | 66 (73.3%) | 62 (68.9%) |
| Ever | 4 (40.0%) | 2 (20.0%) | 2 (20.0%) | 30 (41.1%) | 31 (30.1%) | 45 (43.7%) | 24 (26.7%) | 28 (31.1%) |
| **Drinking status** | | | | | | | | |
| Never | 5 (50.0%) | 7 (70.0%) | 6 (60.0%) | 65 (89.0%) | 89 (86.4%) | 82 (79.6%) | 81 (90.0%) | 78 (86.7%) |
| Ever | 5 (50.0%) | 3 (30.0%) | 4 (40.0%) | 8 (11.0%) | 14 (13.6%) | 21 (20.4%) | 9 (10.0%) | 12 (13.3%) |
| **Family history of GC** | | | | | | | | |
| No | 9 (90.0%) | 9 (90.0%) | 8 (80.0%) | 71(97.3%) | 100 (97.1%) | 96 (93.2%) | 88 (97.8%) | 84 (93.3%) |
| Yes | 1 (10.0%) | 1 (10.0%) | 2 (20.0%) | 2 (2.7%) | 3 (2.9%) | 7 (6.8%) | 2 (2.2%) | 6 (6.7%) |
| **Tumor size (cm)** | | | | | | | | |
| < 5 | _ | 8 (80.0%) | 5 (50.0%) | _ | 72 (69.9%) | 54 (52.4%) | 47 (52.2%) | 56 (62.2%) |
| ≥ 5 | _ | 2 (20.0%) | 5 (50.0%) | _ | 31 (30.1%) | 49 (47.6%) | 43 (47.8%) | 34 (37.8%) |
| **Tumor location** | | | | | | | | |
| Upper third stomach | _ | 1 (10.0%) | 3 (30.0%) | _ | 22 (21.4%) | 31 (30.1%) | 25 (27.8%) | 19 (21.1%) |
| Middle third stomach | _ | 4 (40.0%) | 2 (20.0%) | _ | 58 (56.3%) | 39 (37.9%) | 36 (40.0%) | 43 (47.8%) |
| Lower third stomach | _ | 5 (50.0%) | 5 (50.0%) | _ | 23 (22.3%) | 33 (32.0%) | 29 (32.2%) | 28 (31.1%) |
| **Cell differentiation** | | | | | | | | |
| Well | _ | 3 (30.0%) | 1 (10.0%) | _ | 31 (30.1%) | 25 (24.3%) | 38 (42.2%) | 32 (35.6%) |
| Moderate | _ | 4 (40.0%) | 5 (50.0%) | _ | 47 (45.6%) | 37 (35.9%) | 36 (40.0%) | 27 (30.0%) |
| Poor | _ | 3 (30.0%) | 4 (40.0%) | _ | 25 (24.3%) | 41 (39.8%) | 16 (17.8%) | 31 (34.4%) |
| **Depth of invasion** | | | | | | | | |
| T1 | _ | 2 (20.0%) | 1 (10.0%) | _ | 21 (20.4%) | 11 (10.7%) | 20 (22.2%) | 13 (14.4%) |
| T2 | _ | 4 (40.0%) | 2 (20.0%) | _ | 36 (35.0%) | 29 (28.2%) | 31 (34.4%) | 24 (26.7%) |
| T3 | _ | 3 (30.0%) | 4 (40.0%) | _ | 32 (31.1%) | 31 (30.1%) | 30 (33.3%) | 26 (28.9%) |
| T4 | _ | 1 (10.0%) | 3 (30.0%) | _ | 14 (13.6%) | 32 (31.1%) | 9 (10.0%) | 27 (30.0%) |
| **N category** | | | | | | | | |
| N0 | _ | 10 (100.0%) | _ | _ | 103 (100.0%) | _ | 90 (100.0%) | _ |
| N1 | _ | _ | 4 (40.0%) | _ | _ | 27 (26.2%) | _ | 31 (34.4%) |
| N2 | _ | _ | 4 (40.0%) | _ | _ | 46 (44.7%) | _ | 36 (40.0%) |
| N3 | _ | _ | 2 (20.0%) | _ | _ | 30 (29.1%) | _ | 23 (25.6%) |
| **Stage** | | | | | | | | |
| I | _ | 6 (60.0%) | 1 (10.0%) | _ | 57 (55.3%) | 6 (5.8%) | 58 (64.4%) | 8 (8.9%) |
| II | _ | 3 (30.0%) | 4 (40.0%) | _ | 40 (38.8%) | 34 (33.0%) | 29 (32.2%) | 26 (28.9%) |
| III | _ | 1 (10.0%) | 5 (50.0%) | _ | 6 (5.8%) | 63 (61.2%) | 3 (3.33%) | 56 (62.2%) |
